# Propagation-Invariant Space–Time Plasmonic Pulse in Subwavelength MIM Waveguide

**DOI:** 10.3390/nano14050425

**Published:** 2024-02-26

**Authors:** Eui-Soo Cho, Seung-Yeol Lee

**Affiliations:** School of Electronic and Electrical Engineering, College of IT Engineering, Kyungpook National University, Daegu 41566, Republic of Korea; dmltn2023@knu.ac.kr

**Keywords:** surface plasmon polaritons, metal-insulator-metal plasmonic waveguide, space–time wave packets, diffraction-free beams, propagation-invariant wave packets

## Abstract

The metal-insulator-metal (MIM) plasmonic waveguide has been highly anticipated for confining and guiding surface plasmon polaritons (SPPs) on the subwavelength scale. However, perennial drawbacks such as a short propagation length and an unbounded transverse field have set limits on the use of the MIM waveguide in various applications. Herein, diffraction- and dispersion-free MIM modes are synthesized by using space–time wave packets (STWPs) and are therefore referred to as space–time MIM (ST-MIM) waveguide modes. Compared to a Gaussian pulse of the same duration and spectral bandwidth, the ST-MIM demonstrates enhanced propagation lengths of about 2.4 times for the symmetric mode and about 6.3 times for the antisymmetric mode. In the simulations, the ST-MIMs are confined in all transverse dimensions, thereby overriding the diffraction limits. In addition, the group velocities of the ST-MIMs can be arbitrarily designed, which makes it possible to synchronize the pulse propagation speeds of the symmetric and antisymmetric MIM modes.

## 1. Introduction

The use of surface plasmon polaritons (SPPs) to guide light waves within the subwavelength scale has been extensively researched in the field of nanophotonics [1,2,3]. Such plasmonic waveguides have been used for various applications, such as plasmonic sensors, near-field scanning optical microscopy (NSOM), integrated optical systems, and interconnections between plasmonic and photonic waveguides [4]. Various geometries of plasmonic waveguides, including a single interface [1,2,3,5] as well as dual metal-insulator-metal (MIM) [6], insulator-metal-insulator (IMI) [7], and hybrid layers via multilayered dielectrics [8,9], have been studied for manipulating SPPs within the nanometer scale. Among these geometries, the MIM waveguide has been considered one of the most promising routes for such purposes due to the extremely high confinement, strong field enhancement, and no cutoff frequency characteristics of the fundamental MIM mode ($s_{MIM}$), which has a symmetric transverse magnetic-field profile [10,11,12]. In addition, the higher-order plasmonic mode, which has an antisymmetric transverse magnetic-field profile and is often referred to as the antisymmetric MIM plasmonic mode ($a_{MIM})$, has a great potential to obtain extraordinary characteristics such as light trapping [11] and switchable directional coupling of SPPs [13,14]. However, due to the one-dimensionally (1D) stacked geometry, analytic modal solutions of the MIM waveguide result in an infinitely distributed field along the transverse direction. In other words, the MIM modes are only confined along the metal-gap direction and are fully spread along the parallel-to-film direction, thus making them unsuitable for highly confined plasmonic sensors and integrated optical applications. Therefore, two-dimensionally (2D) confined plasmonic waveguides such as V-shaped groove [15], metal stripe [16,17], waveguides with nanoparticles [18,19] and slot-type waveguides [20] have been developed. However, these 2D-confined plasmonic waveguides have radical drawbacks regarding their modal characteristics, such as the existence of a cutoff frequency and relatively complicated nanofabrication in comparison to simple stacked structures like MIM waveguides.

With respect to the light source for the 1D stacked MIM waveguide, a simple solution is to confine the optical field along the transverse direction by forming a Gaussian profile along that direction. Moreover, temporal confinement is also essential for an optical communications system, which usually uses a pulsed light source, so the Gaussian pulse is one of the simplest solutions. However, a large amount of diffraction and rapid dispersion of the Gaussian pulse might be serious problems and may be even more severe for the $a_{MIM}$ than for the $s_{MIM}$. To address these issues, various non-diffractive beams, such as the Airy or Bessel beam have been considered [21,22]. However, if temporal restrictions are applied, these also have limited characteristics such as losing non-diffracting property and requirement of at least two transverse spatial dimensions and the relatively strong side lobes of the Bessel beam and the curved trajectory of the Airy beam can be additional drawbacks.

Therefore, to achieve propagation-invariant (diffraction- and dispersion-free) pulse characteristics, the MIM waveguide should be carefully designed to satisfy certain relationships in the spatiotemporal domain. In various recent studies, Abouraddy et al. have reported analytic solutions for achieving propagation-invariant light sheets in free space [23], photonic waveguides [24,25], and single-interface SPPs [26] via a unique approach involving the combination of an appropriate wave vector and optical frequency to obtain so-called “space–time wave packets” (STWPs). These STWPs can be designed by choosing an appropriate trajectory, as determined by the intersection of a spectral plane with the dispersion surface (i.e., the revolution surface of the dispersion curve). The shape of the intersection curve can be elliptical, hyperbolic, or parabolic, and the group velocity of the wave packet can be negative. In all cases, these STWPs have maintained non-diffractive and non-dispersive properties and can be applied to modes in any planar structure by careful design of the 2D spatiotemporal profile. By virtue of these characteristics, the STWPs provide a potential new light source solution for the MIM waveguide. Nevertheless, there is still no research aimed at understanding the detailed performance of STWPs designed for MIM plasmonic modes. It is anticipated that such an investigation might reveal the unique features of the STWP, such as the ability to remove modal dispersion from the pulse propagation in the MIM waveguide.

In the present study, diffraction- and dispersion-free MIM modes are synthesized by using space–time wave packets (STWPs) and are therefore referred to as space–time MIM (ST-MIM) waveguides. Further, the advantages of the ST-MIM relative to the conventional Gaussian pulse in the MIM waveguide are investigated. The results indicate that the propagation length increases considerably in both the $s_{MIM}$ and $a_{MIM}$. Moreover, the propagation length of the ST-MIM pulse formed by the $a_{MIM}$ is longer than that of the Gaussian pulse formed by the $s_{MIM}$, in contrast to the general rule-of-thumb that the $a_{MIM}$ has a shorter propagation length than the $s_{MIM}$ in most cases. Moreover, the confinement of the ST-MIM is also extremely high in all dimensions, along with dispersion- and diffraction-free characteristics. Finally, identical values for the $s_{MIM}$ and $a_{MIM}$ can be achieved by appropriately designing the trajectory curve. Through this approach, modal dispersion-free pulse propagation through the MIM plasmonic waveguide is demonstrated.

## 2. ST-MIM Design Principle

Herein, the ST-MIM and Gaussian pulses are intuitively modeled via analytical calculations using MATLAB. The carrier wavelength is appropriately selected at 650 nm to avoid the cutoff condition of $a_{MIM}$, and to consider the potential use of plasmonic applications such as near-field-scanning microscopes. The MIM waveguide consists of Ag-SiO_2_-Ag planar stacks, and the detailed parameters are summarized in Table 1. The parameters of Table 1 are identically designed for both the Gaussian pulse and ST-MIM, except for the wavelength range. The difference in wavelength range between the Gaussian pulse and ST-MIM stems from the difference in spectral distribution of each mode in spectral-space $\left(k_{z},\;k_{y},\;\frac{\omega }{c_{0}}\right)$. The spectral distribution of a Gaussian pulse has a patch shape that is attached to the dispersion surface so that the carrier wavelength is located at the center of the wavelength range. On the other hand, the spectral distribution of ST-MIM has a convex parabolic shape so that the carrier wavelength becomes the maximum wavelength because of the design principle of ST-MIM. The spectral tilt angle indicates the angle between the spectral plane $P\left(\varphi_{ST}\right)$, which is defined in spectral-space $\left(k_{z},\;k_{y},\;\frac{\omega }{c_{0}}\right)$ and the $\frac{\omega }{c_{0}}$-axis [23].

**Figure 1:**
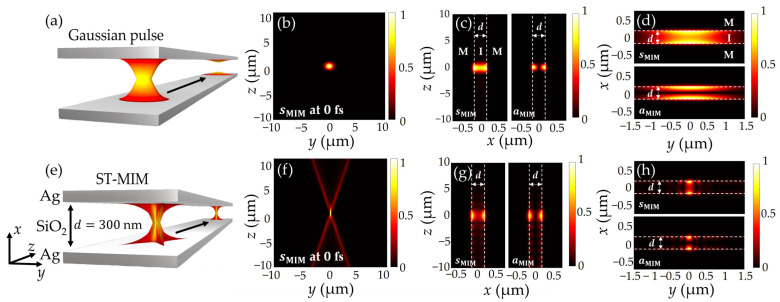
Conceptual images and normalized ${\left|H_{y}\right|}^{2}$ distributions in all transverse planes comparing the propagation performances of (**a**–**d**) the conventional MIM Gaussian pulse and (**e**–**h**) the ST-MIM pulse. Here, each pulse is initially focused along the transverse (*y*) direction at *z* = 0, is structurally confined along the gap (*x*) direction, and propagates along the *z* direction. (**a**,**e**) The conceptual images, (**b**,**f**) the normalized ${\left|H_{y}\right|}^{2}$ distributions of the $s_{MIM}$ in the *yz*-plane (*x* = 0); (**c**,**g**) the normalized ${\left|H_{y}\right|}^{2}$ distributions of the $s_{MIM}$ (left) and $a_{MIM}$ (right) separately positioned in the *xz*-plane (*y* = 0); (**d**,**h**) the normalized ${\left|H_{y}\right|}^{2}$ distributions of the $s_{MIM}$ (top) and $a_{MIM}$ (bottom) separately positioned in the *xy*-plane (*z* = 0).

First, the confinement characteristics of the ST-MIM are compared with those of a conventional Gaussian pulse with the same pulse duration and spectral bandwidth (Figure 1). The conceptual image in Figure 1a indicates that the Gaussian pulse rapidly decays along the propagation direction due to its diffraction and dispersion characteristics. Further, under the focused condition, the corresponding normalized ${\left|H_{y}\right|}^{2}$ distribution formed by the $s_{MIM}$ along the transverse (*yz*) plane has a full width half maximum (FWHM) of 1.16 μm along the transverse (*y*) direction (Figure 1b). In addition, the confinement profiles of the Gaussian pulses along the MIM core are shown separately for the $s_{MIM}$ (left) and $a_{MIM}$ (right) in Figure 1c. Here, the $s_{MIM}$ exhibits a strong field amplitude at the center of the insulator (x=0), whereas the $a_{MIM}$ has zero amplitude due to the antisymmetric phase profile. Similarly, the normalized ${\left|H_{y}\right|}^{2}$ along transverse directions are shown separately for the $s_{MIM}$ (top) and $a_{MIM}$ (bottom) in Figure 1d, where the field distribution is similar to that of Figure 1c, and reveals the difference in the field profile at the center of the insulator (x=0).

By contrast, the ST-MIM maintains its shape during propagation, thus exhibiting a propagation-invariant field profile (Figure 1e), as verified in the following section. Moreover, the normalized ${\left|H_{y}\right|}^{2}$ distribution formed by the $s_{MIM}$ along the *y*-direction has an FWHM of 216 nm (Figure 1f). Furthermore, Figure 1g indicates that both modes of the ST-MIM are similarly confined to those of the Gaussian pulse (Figure 1c). However, the electromagnetic fields of the proposed ST-MIM are much more strongly confined in the *xy*-plane, giving values of 216 and 168 nm for the $s_{MIM}$ and $a_{MIM}$, respectively (Figure 1h), compared to 1.16 μm for both mode of the Gaussian pulse (Figure 1d). Therefore, the proposed ST-MIM pulse can provide subwavelength scale confinement for all transverse directions. Moreover, it will not suffer any pulse broadening, as verified in Section 3. To obtain the fields of MIM mode, the Maxwell equations are solved with boundary conditions which consist of metal-insulator-metal. From these solutions, TM mode ($E_{x}$, $H_{y}$, $E_{z}$) is obtained [29]. Then, spectral components are obtained by solving following equations as mentioned below.

**Figure 2:**
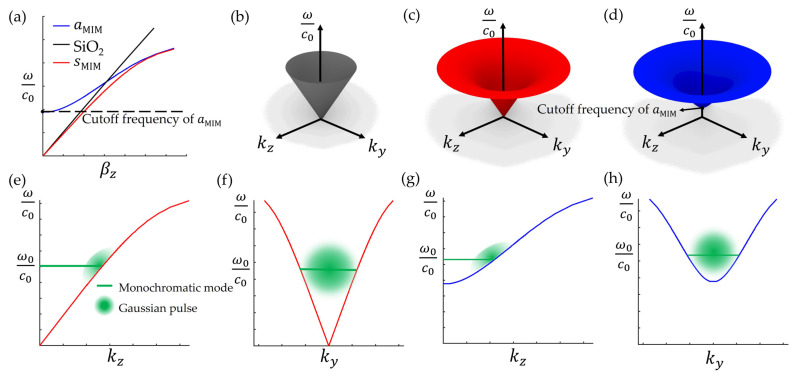
(**a**) The dispersion relationships of the $s_{MIM}$ and $a_{MIM}$ and a light line in homogeneous SiO_2_. (**b**) The light-cone of homogenous SiO_2_. (**c**,**d**) The dispersion surfaces of (**c**) the $s_{MIM}$ and (**d**) the $a_{MIM}$ drawn in $\left(k_{z},\;k_{y},\frac{\omega }{c_{0}}\right)$ space. (**e**,**f**) The spectral projections for the monochromatic mode and the Gaussian pulse of the $s_{MIM}$ drawn on (**e**) the $\left(k_{z},\frac{\omega }{c_{0}}\right)$-plane and (**f**) the $\left(k_{y},\frac{\omega }{c_{0}}\right)$-plane. (**g**,**h**) Similar diagrams to those in (**e**,**f**), but for the $a_{MIM}$.

To compose the ST-MIM pulse, the dispersion relationship of the MIM waveguide is first considered, as shown in Figure 2. Here, the propagation axis is defined as the *z*-axis, so the dispersion curve is obtained from the relationship between the *z*-directional complex propagation constant ($\beta_{z}$) and the wavevector ($\frac{\omega }{c_{0}})$, where ω is the frequency and $c_{0}$ is the speed of light in free space. The black line in Figure 2a illustrates the dispersion relationship for the plane wave in homogeneous SiO_2_, i.e., $\frac{\omega }{c_{0}}=\frac{\beta_{z}}{n_{{SiO}_{2}}}$, where $n_{{SiO}_{2}}$ is the refractive index of SiO_2_. The light-cone of homogeneous SiO_2_ can be defined as the revolution surface of the dispersion relationship drawn in spectral space $\left(k_{z},\;k_{y},\;\frac{\omega }{c_{0}}\right),$ as illustrated in Figure 2b. Meanwhile, the red and blue lines in Figure 2a represent the dispersion curves of the $s_{MIM}$ and $a_{MIM}$, which can be drawn by determining propagation constant ($\beta_{\xi }$) where ξ is the mode ($s_{MIM}$ or $a_{MIM}$). In order to determine $\beta_{\xi }$ of the $s_{MIM}$ and $a_{MIM}$, we should obtain *x*-directional complex wavenumber ($k_{x,{SiO}_{2}}$ and $k_{x,Ag}$) by numerically solving Equations (1) and (2), respectively:

(1) $$\tanh\left(\frac{-jk_{x,{SiO}_{2}}d}{2}\right)=-\frac{\varepsilon_{{SiO}_{2}}k_{x,Ag}}{\varepsilon_{Ag}k_{x,{SiO}_{2}}},$$

(2) $$\coth\left(\frac{-jk_{x,{SiO}_{2}}d}{2}\right)=-\frac{\varepsilon_{{SiO}_{2}}k_{x,Ag}}{\varepsilon_{Ag}k_{x,{SiO}_{2}}},$$

where $\varepsilon_{{SiO}_{2}}$ and $\varepsilon_{Ag}$ are the electrical complex permittivity of SiO_2_ and Ag, respectively, and d is the thickness of the MIM core.

The *z*-directional complex propagation constant ($\beta_{\xi }$) is defined by Equation (3):

(3) $${\beta_{\xi }}^{2}=\varepsilon_{r,L}{\left(\frac{\omega_{0}}{c_{0}}\right)}^{2}-{k_{x,L}}^{2},$$

where $\varepsilon_{r}$ is the relative electrical permittivity, L is the layer material (which can be SiO_2_ or Ag), $\omega_{0}$ is the carrier (angular) frequency, $k_{0}$ is the carrier wavenumber in free space corresponding to $\frac{\omega_{0}}{c_{0}}$, and $\beta_{\xi }$ is the propagation constant of the relevant MIM mode, which corresponds to $\beta_{\xi }=n_{\xi }k_{0}$. Therefore, $n_{\xi }$ can be found by simultaneously solving Equations (1)–(3).

By considering the composition of the wavevectors for specific MIM modes, $\beta_{\xi }$ can be decomposed into two complex wavenumber components, $k_{y}$ and $k_{z}$, as given by Equation (4):

(4) $${\beta_{\xi }}^{2}={k_{y}}^{2}+{k_{z}}^{2}.$$

As with the single-interface SPP mode, the dispersion relationship of the $s_{MIM}$ has no cutoff frequency. However, that of the $a_{MIM}$ has a cutoff frequency and generally exhibits a slower group velocity than that of the $s_{MIM}$. The dispersion surfaces of both modes in spectral space $\left(k_{z},\;k_{y},\;\frac{\omega }{c_{0}}\right)$ are shown in Figure 2c,d, respectively. In addition, the cross-sectional views are plotted in Figure 2e,f for the $s_{MIM}$ and Figure 2g,h for the $a_{MIM}$.

Using these dispersion surfaces, the angular-spectral distributions for various beams and pulses can be described. For example, any monochromatic beam formed by the $s_{MIM}$ or $a_{MIM}$ can be expressed as the circular intersection curve of each dispersion surface and iso-frequency $\omega =\omega_{0}$ plane, as shown by the green lines in Figure 2e–h. In addition, the spectral distributions of the Gaussian pulse exhibit a patch profile attached to the relevant dispersion surface, as indicated by the green circular shades.

**Figure 3:**
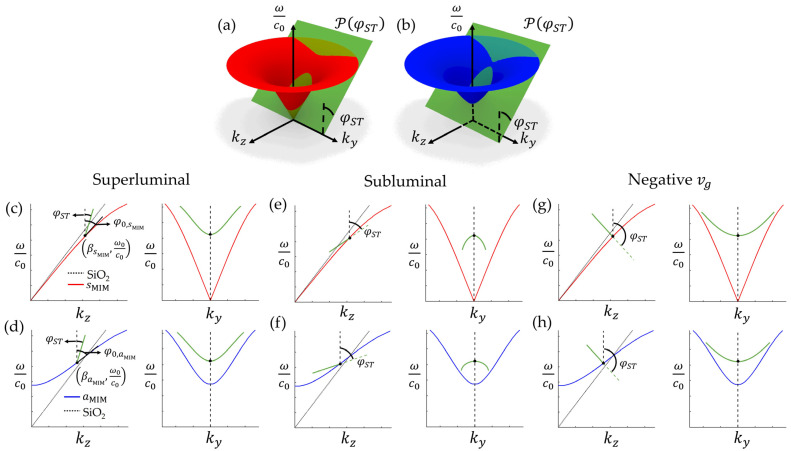
(**a**,**b**) Conceptual images for extracting the spatiotemporal relationship of the ST-MIM by plotting the intersection of the MIM light-cone of (**a**) the $s_{MIM}$ and (**b**) the $a_{MIM}$ with the spectral plane $P\left(\varphi_{ST}\right)$ in $\left(k_{z},\;k_{y},\frac{\omega }{c_{0}}\right)$ space. (**c**,**d**) The spectral projections of a superluminal ST-MIM onto the $\left(k_{z},\frac{\omega }{c_{0}}\right)$-plane (left) and the $\left(k_{y},\frac{\omega }{c_{0}}\right)$-plane (right) for (**c**) the $s_{MIM}$ and (**d**) the $a_{MIM}$. Here, $\varphi_{0,s_{MIM}}$ and $\varphi_{0,a_{MIM}}$ are tangential angles of the $s_{MIM}$ and $a_{MIM}$, respectively. (**e**–**h**) Similar diagrams for a subluminal ST-MIM (**e**,**f**) and a negative $\nu_{g}$ ST-MIM (**g**,**h**).

In the case of the ST-MIM, the angular-spectral distribution should satisfy the expression for the spectral plane $P\left(\varphi_{ST}\right)$, as given in Equation (5):

(5) $$k_{z}=\beta_{\xi }+\left(\frac{\omega -\omega_{0}}{c_{0}}-k_{0}\right)\tan\left(\varphi_{ST}\right).$$

where $\varphi_{ST}$ is the spectral tilt angle that finally determines the group velocity of the ST-MIM pulse. Then, the intersection of the relevant dispersion surface with $P\left(\varphi_{ST}\right)$ indicates the angular spectral distribution of the ST-MIM pulse formed by either the $s_{MIM}$ or the $a_{MIM}$, as shown in Figure 3a,b. Here, by integrating each plane wave with the wavenumber $k_{z}$ from Equation (5), $k_{y}$ from Equation (4), and their corresponding frequency ω. The projection curves of the angular spectral distribution on the $\left(k_{z},\;\frac{\omega }{c_{0}}\right)$- and $\left(k_{y},\;\frac{\omega }{c_{0}}\right)$-plane indicate that the ST-MIM does not suffer any dispersion or diffraction during propagation due to its linear and one-to-one relationship [30]. The dispersion-free characteristic stems from the linear property, which maintains an identical tangential value. Also, the diffraction-free characteristic can be achieved by a one-to-one relationship between $k_{y}$ and ω, in which the diffracting phase factor resulting from $k_{y}$ is compensated by the phase factor from ω.

Since there is a degree of freedom for choosing the value of $\varphi_{ST}$, the group velocity ($v_{g}$) of the ST-MIM can also be arbitrarily designed. This is given by the relationships $\nu_{g}=\frac{\Delta \omega }{\Delta \beta }=\frac{\omega -\omega_{0}}{k_{z}-\beta_{\xi }}=c_{0}\cot\varphi_{ST}$, in Equation (5). For example, when $P\left(\varphi_{ST}\right)$ is tangential to the dispersion surfaces at $\omega =\omega_{0}$, the group velocity of the ST-MIM has the same value as that of the normally propagating simple MIM mode. This condition is defined herein as ‘luminal,’ and it satisfies the expressions $\varphi_{ST}=\varphi_{0,\;s_{MIM}}$ for the $s_{MIM}$ and $\varphi_{ST}=\varphi_{0,\;a_{MIM}}$ for the $a_{MIM}$. With the appropriate choice of $\varphi_{ST}$, the $v_{g}$ can be designed to have superluminal, subluminal, or even negative values. In both modes, the superluminal ST-MIM has $\varphi_{ST}$ values in the range of $0<\varphi_{ST}<\varphi_{0}$, corresponding to hyperbolic spectral projections, as shown for the $s_{MIM}$ and $a_{MIM}$ in Figure 3c,d, respectively. Conversely, the subluminal ST-MIM has values of $\varphi_{0}<\varphi_{ST}<90^{{}^{\circ}},$ corresponding to elliptical spectral projections, as shown in Figure 3e,f for the $s_{MIM}$ and $a_{MIM}$, respectively. The $\varphi_{ST}$ range for negative $v_{g}$ can also be designed by setting $90^{{}^{\circ}}<\varphi_{ST}<180^{{}^{\circ}}$, giving the spectral projections shown in Figure 3g,h.

**Figure 4:**
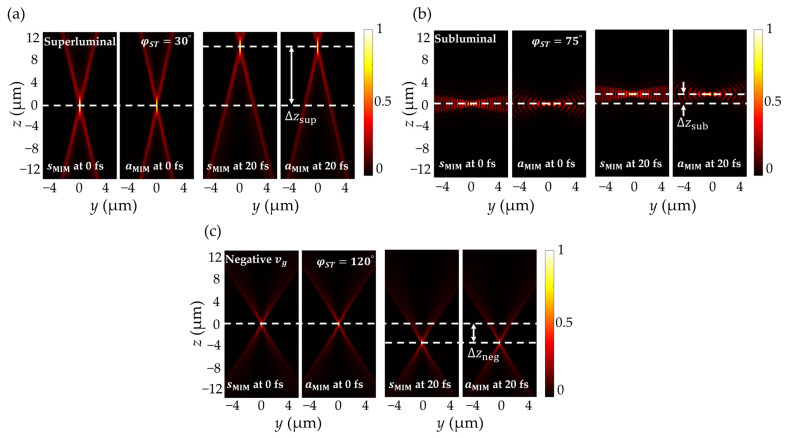
The normalized ${\left|H_{y}\right|}^{2}$ plots of the $s_{MIM}$ and ${\left|E_{z}\right|}^{2}$ plots of the $a_{MIM}$ for (**a**) the superluminal, (**b**) the subluminal, and (**c**) the negative group velocity ST-MIM at propagation times of t=0 (left) and t=20 fs (right) along the *yz*-plane. Here, the spectral tilt angle ($\varphi_{ST}$) values are $30^{\circ }$, $75^{\circ }$, and $120^{\circ }$, respectively, and the corresponding ∆z values are 10.56 μm $\left(∆z_{sup}\right)$, 1.6 μm $\left(∆z_{sub}\right)$, and −3.45 μm $\left(∆z_{neg}\right)$, respectively.

The propagation properties of superluminal, subluminal, and negative group velocity ST-MIMs in both the $s_{MIM}$ and $a_{MIM}$ are compared by the field distributions of the ST-MIMs at t=0 (left) and at t=20 fs (right) in Figure 4. Here, the $\varphi_{ST}$ values of the superluminal, subluminal, and negative group velocity ST-MIMs are set to $30^{\circ }$, $75^{\circ }$, and $120^{\circ }$, respectively. Accordingly, the group velocity of each ST-MIM is $5.18\times 10^{8}$, 8.01 $\times \;10^{7}$, and $-1.73\times 10^{8}$ m/s, respectively, and the corresponding propagation distances are $\Delta z_{sup}=10.56$ μm, $\Delta z_{sub}=1.6$ μm, and $\Delta z_{neg}=-3.45$ μm. Since the information velocity of light can be different from the group velocity [31,32], the group velocity of superluminal ST-MIM can exceed the speed of light in free-space. In detail, the photon located at the pulse center of the superluminal ST-MIM at certain time does not move from the pulse center of the previous time but from the X-shaped side-lobe, which is already located much forward than the pulse center. Therefore, without violating the law of physics, superluminal group velocity can be achieved, but that does not mean the information carried by ST-MIM can exceed the absolute speed of light. Since the group velocities only depend on $\varphi_{ST}$, synchronization of the group velocities for the two MIM modes can be obtained. Also, because the angular-spectral distribution of the ST-MIM varies according to $\varphi_{ST}$, the shape of the ST-MIM varies correspondingly.

## 3. The Propagation-Invariant Performance of the ST-MIM

**Figure 5:**
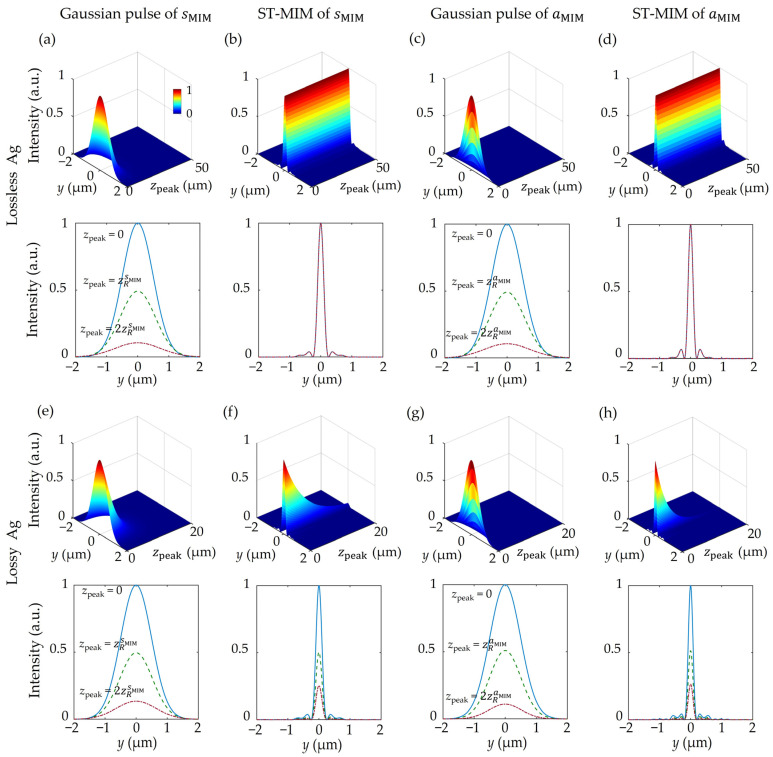
A comparison of the diffraction and dispersion properties of the Gaussian pulse and the ST-MIM without (**a**–**d**) and with (**e**–**h**) ohmic loss due to Ag. Here, the peak envelope cross-sections are plotted along the pulse propagation direction for (**a**,**e**) the $s_{MIM}$ of the Gaussian pulse, (**b**,**f**) the $s_{MIM}$ of the ST-MIM, (**c,g**) the $a_{MIM}$ of the Gaussian pulse, and (**d**,**h**) the $a_{MIM}$ of the ST-MIM.

To verify the non-diffractive and non-dispersive properties of the ST-MIM, the propagation dynamics of the peak envelope cross-section is plotted in Figure 5. In other words, the time-dependent evolution of the center location of the pulse envelope is tracked, and the information is noted as $z_{peak}$. To clearly show the attenuation characteristics, ${\left|H_{y}\right|}^{2}$ of $s_{MIM}$ and ${\left|E_{z}\right|}^{2}$ of $a_{MIM}$ at x=0 are used to plot the peak envelope cross-section. Thus, the diffraction characteristics of a given pulse according to the elapsed time can be clearly observed by plotting the *y*-direction intensity profile along the center of the pulse envelope for each condition of $z_{peak}$, as shown in the 3D color-coded graphs in Figure 5a–h. Meanwhile, the lower graph in each part of figure plots the cross-section of the corresponding upper graph at $z_{peak}=0$, $z_{peak}=z_{R}^{\xi }$ and $z_{peak}=2z_{R}^{\xi }$. Here, $z_{R}^{\xi }$ is the Rayleigh range calculated form the reference Gaussian pulse. In Figure 5a–d, the ohmic loss of each Ag layer is ignored by neglecting the imaginary part of the complex permittivity data in order to focus on the pure diffraction characteristics. Then, Figure 5e–h shows the more practical case in which the ohmic loss of Ag is considered.

Even if there is no ohmic loss, the peak intensity of the $s_{MIM}$ Gaussian pulse decays rapidly due to its diffraction and dispersion, with a $z_{R}^{s_{MIM}}\;of\;3.82$ μm and a propagation length of 7.13 μm (Figure 5a). By contrast, the $s_{MIM}$ of the ST-MIM does not suffer from diffraction and dispersion, and therefore propagates almost infinitely while maintaining its cross-sectional shape at $z_{peak}=0$ (Figure 5b) if a lossless metal is considered. In the case of the $a_{MIM}$, the Gaussian pulse suffers a much faster decay than that of the $s_{MIM}$ due to its stronger diffraction and dispersion, with a $z_{R}^{a_{MIM}}\;of\;0.73\;\mu m$ and a propagation length of 1.24 μm (Figure 5c). However, as with the $s_{MIM}$, the $a_{MIM}$ of the ST-MIM propagates without decaying and maintains a uniform cross-section during the propagation (Figure 5d).

When ohmic loss is considered, the propagation dynamics of the $s_{MIM}$ for the Gaussian pulse exhibit a somewhat shorter Rayleigh range ($z_{R}^{s_{MIM}}=2.55$ μm) and propagation length (5.51 μm) (Figure 5e). However, while the $s_{MIM}$ of the ST-MIM also has a finite propagation length (13.06 μm), this is 2.4 times longer than that of the Gaussian pulse. Moreover, despite the ohmic loss, the $s_{MIM}$ of the ST-MIM exhibits self-similar cross-sections due to its diffraction- and dispersion-free properties (Figure 5f). Meanwhile, the $a_{MIM}$ of the lossy Gaussian pulse exhibits a quite similar performance to that observed in the lossless case, with a $z_{R}^{a_{MIM}}\;of\;0.63$ μm and a propagation length of 1.14 μm (Figure 5g), thereby suggesting that diffraction and dispersion occur rather than ohmic loss. By contrast, the $a_{MIM}$ of the ST-MIM has a significantly better propagation length of 7.18 μm, which is 6.3 times larger than that of the Gaussian case due to propagation-invariance, along with self-similar cross-sections (Figure 5h). Notably, the propagation length of the $s_{MIM}$ is higher than that of the $a_{MIM}$ for the lossy ST-MIM, because the imaginary part of the effective refractive index of the $a_{MIM}$ is generally higher than that of the $s_{MIM}$.

**Figure 6:**
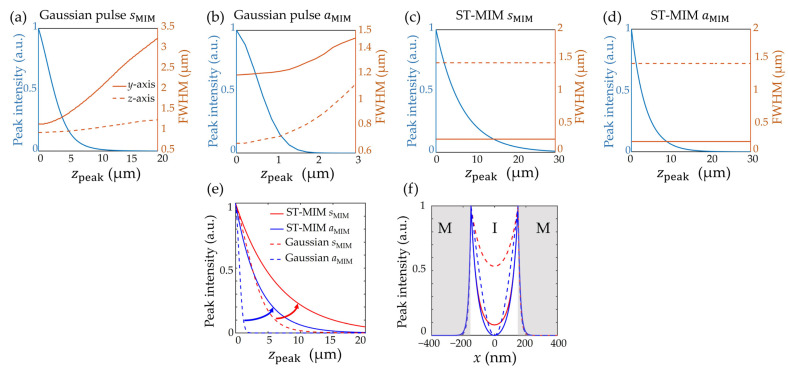
Plots of the peak intensity of the pulse envelope (blue solid lines) and the FWHM along the *y*-axis (orange solid lines) and *z*-axis (orange dashed lines) for (**a**) the $s_{MIM}$ of the Gaussian pulse, (**b**) the $a_{MIM}$ of the Gaussian pulse, (**c**) the $s_{MIM}$ of the ST-MIM, and (**d**) the $a_{MIM}$ of the ST-MIM. (**e**,**f**) The corresponding plots of (**e**) the pulse peak intensity and (**f**) the *x*-directional field profiles.

More detailed data comparing the diffraction-free, dispersion-free, and enhanced propagation performance of the ST-MIM relative to the Gaussian pulse in the MIM waveguide are presented in Figure 6. The peak intensities of the pulse envelope are presented in Figure 6a–d, along with the corresponding FWHM values along the *y*- and *z*-axes, which show the respective beam width and wave packet length during the pulse propagation. Thus, the beam width and wave packet length of both the $s_{MIM}$ and $a_{MIM}$ of the Gaussian pulse are seen to increase rapidly with propagation (Figure 6a,b), thereby indicating severe diffraction and dispersion. This effect appears much stronger for the $a_{MIM}$ than for the $s_{MIM}$ due to the larger group velocity dispersion of the antisymmetric mode. By contrast, the beam width of the ST-MIM for the $s_{MIM}$ and $a_{MIM}$ remain at 216 and 168 nm, respectively, and the wave packet length remains at 1.46 μm and 1.44 μm, respectively, thus suggesting diffraction- and dispersion-free characteristics (Figure 6c,d). Further, the peak intensities for the $s_{MIM}$ and $a_{MIM}$ of both the Gaussian pulses and the ST-MIMs are plotted on the same chart in Figure 6e, clearly demonstrating the improvement in the propagation length of the ST-MIM. The propagation length of the ST-MIM is generally longer than that of the Gaussian pulse for both modes, and it is noteworthy that the $a_{MIM}$ of the ST-MIM exhibits an even longer propagation length than that of the Gaussian pulse $s_{MIM}$. These data imply that the propagation length performance of the $a_{MIM}$ can be improved to a comparable scale to that of the conventional $s_{MIM}$ pulses with the help of the ST-MIM configuration, thereby expanding the usefulness of the $a_{MIM}$ into various plasmonic devices and applications. In addition, the confinement along the MIM gap direction of the ST-MIM is shown in Figure 6f, where the modes are obviously confined in the core insulator layer with a thickness of 300 nm, as with the conventional Gaussian pulse.

**Figure 7:**
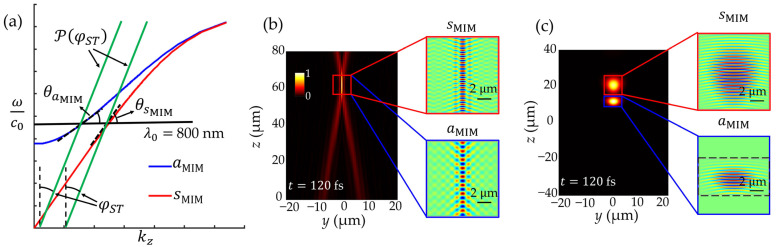
(**a**) The spectral projection of the multimodal ST-MIM onto the $\left(k_{z},\;\frac{\omega }{c_{0}}\right)$-plane, where $\theta_{s_{MIM}}$ and $\theta_{a_{MIM}}$ are the tangential angles at $\lambda_{0}=$ 800 nm for the light-cones of the $s_{MIM}$ and $a_{MIM}$, respectively. (**b**,**c**) The normalized intensity distribution of (**b**) a multimodal ST-MIM pulse (Supplementary Video S1), and (**c**) a multimodal Gaussian pulse (Supplementary Video S2) at t=120 fs, i.e., $I\left(x=0,\;y,z;t=120\;fs\right)$. To emphasize the difference between the multimodal ST-MIM and the multimode Gaussian pulse, the Ag is treated as a lossless material in this simulation.

The enhanced propagation length of the $a_{MIM}$, along with the adjustability of the fixed group velocity, can lead to the full removal of the modal dispersion issue from the MIM waveguide. As with conventional optical fibers, the multimodal photonic/plasmonic waveguide may become desirable for high-bandwidth communication services in integrated photonic circuits [33,34]. In this respect, the implementation of multimodal propagation through conventional MIM pulses while synchronizing the group velocities of both the $s_{MIM}$ and $a_{MIM}$ is a fundamentally difficult task due to the difference in the dispersion relation of each mode, especially in the region around the cutoff frequency. However, due to the tunable group velocity and propagation-invariance of the ST-MIM, it is possible to achieve multimodal MIM pulse propagation with ease and without modal dispersion, as demonstrated in Figure 7. Here, a carrier wavelength of 800 nm is selected in order to demonstrate the synchronization of the group velocity of both modes in the region where a significant difference in group velocity dispersion would occur. As shown in Figure 7a, the group velocity ($\frac{\Delta \omega }{\Delta \beta }$) of the Gaussian $s_{MIM}$ and $a_{MIM}$ pulses are not the same; i.e., $\theta_{s_{MIM}}\neq \theta_{a_{MIM}}$, where $\theta_{s_{MIM}}$ is the angle of the tangential line for the $s_{MIM}$ and $\theta_{a_{MIM}}$ is that for the $a_{MIM}$. By contrast, the spectral tilt angles formed by the $s_{MIM}$ and $a_{MIM}$ of the ST-MIM can be designed to have identical values $\left(\varphi_{ST}=30^{{}^{\circ}}\right)$, thereby resulting in an identical group velocity of $c_{0}\cot\varphi_{ST}$ for both modes. Further, the intensity distributions of the ST-MIM and Gaussian pulses in the *yz*-plane after propagating for t=120 fs are represented in Figure 7b,c, respectively. Here, although the ST-MIMs of both modes are perfectly combined as if just one mode were propagating, the Gaussian pulses of both modes are clearly split due to the faster group velocity of the $s_{MIM}$ relative to that of the $a_{MIM}$. Because the slope of the line from the origin to the point $\left(\frac{\omega_{0}}{c_{0}},\;k_{z}\right)$ of the antisymmetric mode is higher than that of the symmetric mode, the phase velocity of the antisymmetric mode is faster than that of the symmetric mode in both the ST-MIM and the Gaussian pulse, as shown by the real value distributions in the red and blue insets of Figure 7b,c.

The above results demonstrate the ability of the proposed ST-MIM to provide the numerous benefits of the previously reported STWPs, including tunability (acceleration or deceleration) of the group velocity [35,36], self-healing [37], arbitrary dispersion profiles [38,39], incoherent broadband fields [40], and omni-resonance [41].

## 4. Conclusions

Herein, a spatiotemporally correlated wave packet was proposed for a metal-insulator-metal (MIM) plasmonic waveguide, which was designated as the ST-MIM. The ST-MIM was constructed by synthesizing MIM modes with various frequency (ω) and wavenumber ($k_{x},k_{y},k_{z}$ values, which were extracted from the spatiotemporally correlated equations. The ST-MIM exhibited diffraction-free and dispersion-free properties, thus leading to propagation-invariant performance, customizable group velocity, and other space–time wave packet (STWP) properties. Compared to Gaussian pulses, the propagation-invariance of the ST-MIM increases the propagation length by about 2.4 times and 6.3 times for the $s_{MIM}$ and $a_{MIM}$, respectively. In particular, the increased propagation length of the $a_{MIM}$ and the tunability of the group velocity in the ST-MIM configuration enable the implementation of MIM pulse propagation with synchronized group velocity, which has been quite difficult to achieve with conventional Gaussian pulses.

## Figures and Tables

**Table 1:** The simulation parameters for the ST-MIM and Gaussian pulses.

Parameter	Value	
	Figure 1, Figure 2, Figure 3, Figure 4, Figure 5 and Figure 6	Figure 7
Carrier wavelength $\left(\lambda_{0}\right)$	650 nm	800 nm
Relative permittivity of insulator SiO_2_ $\left(\varepsilon_{r,{SiO}_{2}}\right)$	Extracted from [27]	
Thickness of insulator	300 nm	
Relative permittivity of metal Ag $\left(\varepsilon_{r,Ag}\right)$	Extracted from [28]	
Effective refractive index of symmetric mode at $\lambda_{0}$ $\left(n_{eff,s_{MIM}}\right)$	1.6216+j0.00793	1.5917+j0.00452
Effective refractive index of antisymmetric mode at $\lambda_{0}$ $\left(n_{eff,a_{MIM}}\right)$	1.3525+j0.01442	1.0475+j0.01123
Pulse duration	5 fs	
Spectral bandwidth	0.2 PHz	
Wavelength range $(\lambda_{min}$–$\lambda_{max}$)	Gaussian	
	534–831 nm	766–837 nm
	ST-MIM	
	453–650 nm	734–800 nm
Spectral tilt angle $\left(\varphi_{ST}\right)$	$45^{{}^{\circ}}$	30°

## Data Availability

The data presented in this study are available on request.

## Supplementary Materials

The following supporting information can be downloaded at https://www.mdpi.com/article/10.3390/nano14050425/s1: propagation video for the multimodal ST-MIM (Video S1); propagation video for the multimodal Gaussian pulse (Video S2).
